# Supplementary material for: Expression of Lineage Transcription Factors Identifies Differences in Transition States of Induced Human Oligodendrocyte Differentiation
Source: Cells. 2022 Jan 11;11(2):241. doi: 10.3390/cells11020241 (PMC8773672; doi:10.3390/cells11020241)
Supplement: Supplementary file 1 [file cells-11-00241-s001.zip › cells-1542523-supplementary/Raabe et al._iOPC-iOL_supplemental information.pdf]

Inventory of Supplemental Information

- Supplemental Figures
- Supplemental Tables
- Supplemental References

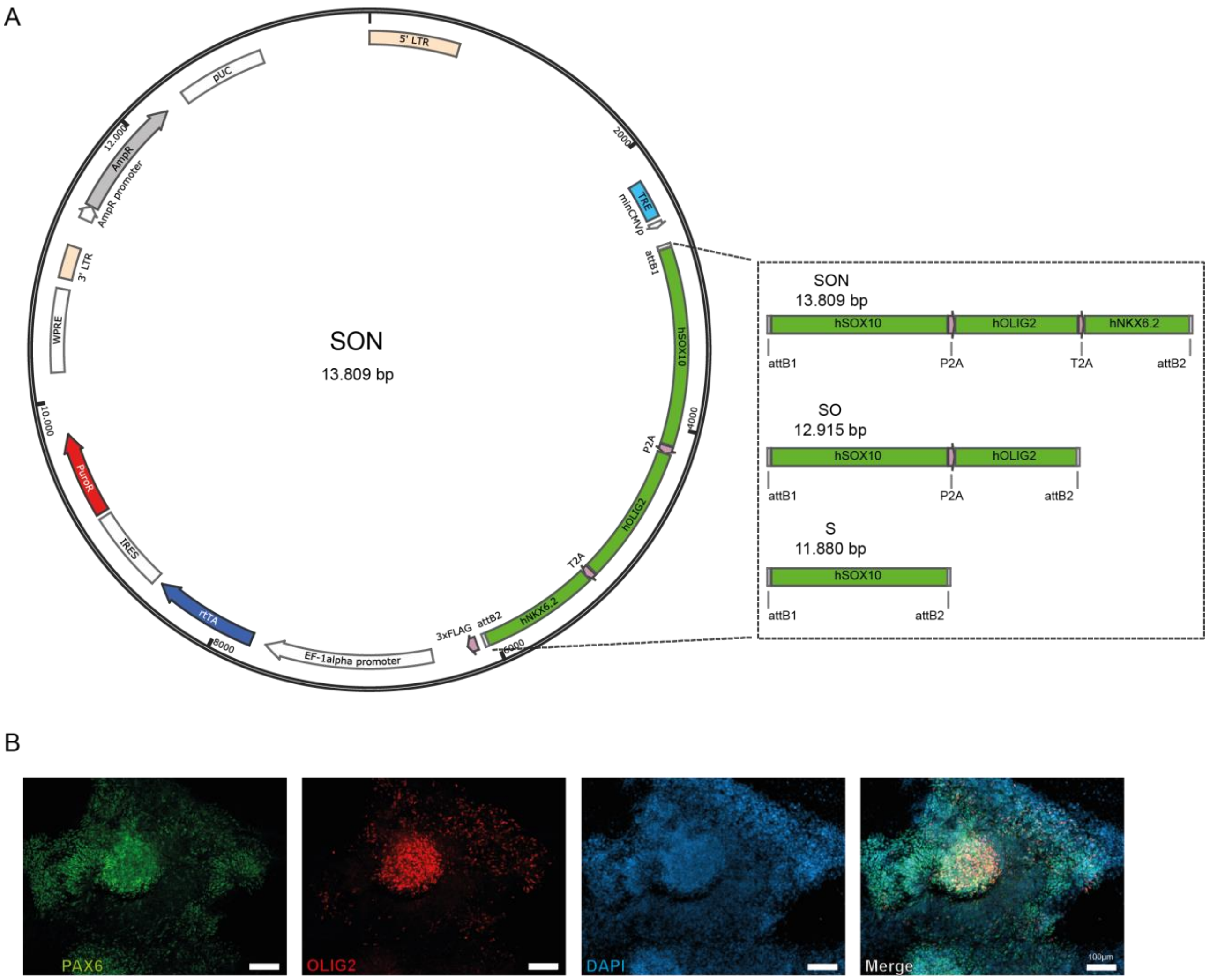

Figure S1. Detailed illustration of applied lentiviral construct and neural induction. Related to Figure 1.

(A) Schematic overview of the applied lentiviral vector containing constitutive expression units for the reversed tetracycline transactivator (rtTA) and puromycin N-acetyl-transferase (PuroR) and a tetracycline response element (TRE) for the doxycycline-dependent overexpression of the TFs. Magnification illustrates the different TFs combinations using SON, SO and S that are linked by the self-cleavage sites P2A and T2A. Plasmid maps of the constructs (S, SO, SON) are available in the supplemental files.

(B) Representative images of $PAX6^{+}$ (green), $OLIG2^{+}$ (red) neural precursor cells (NPC) 12 days after neural induction. Scale bar: 100µm.

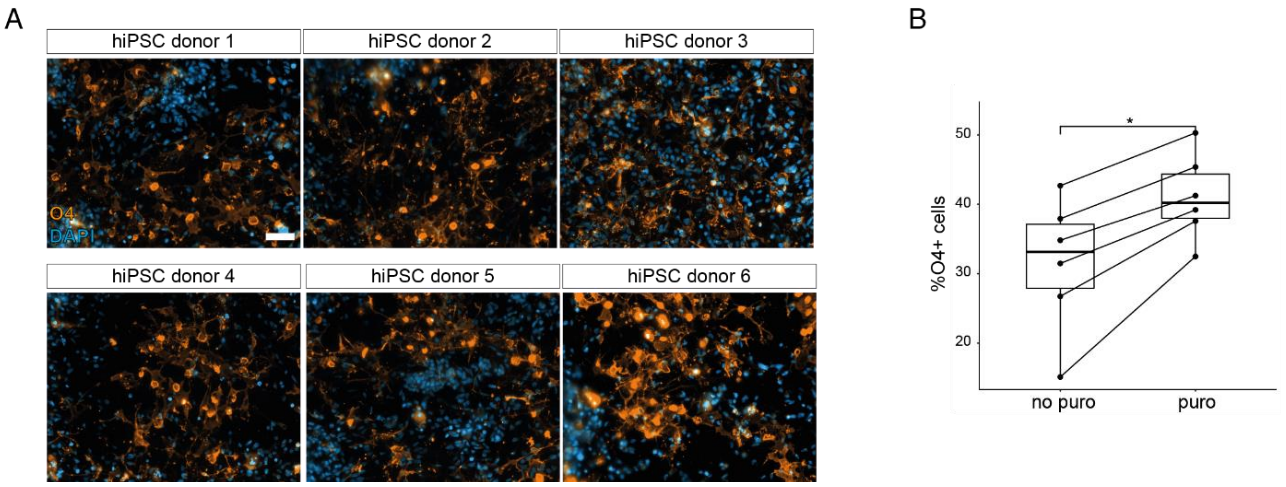

Figure S2. SON-directed differentiation from different hiPSC lines and effect of intermediate selection. Related to Figure 2.

(A) Illustration of $O4^{+}$ cells (orange) from six different hiPSC lines of independent donors 10days of SON-directed differentiation with intermediate puromycin selection (puro). Scale bar: 50µm.

(B) Quantification of $O4^{+}$ cells after 10 days of SON-directed differentiation with and without intermediate puromycin selection between Day+2 and Day+4. 13 analysed fields of view for each condition, Dots correspond to mean percentage of $O4^{+}$ cells per condition. Lines connects both conditions from one hiPSC cell line. Statistical analysis by Wilcoxon signed-rank test, $*p < 0.05$.

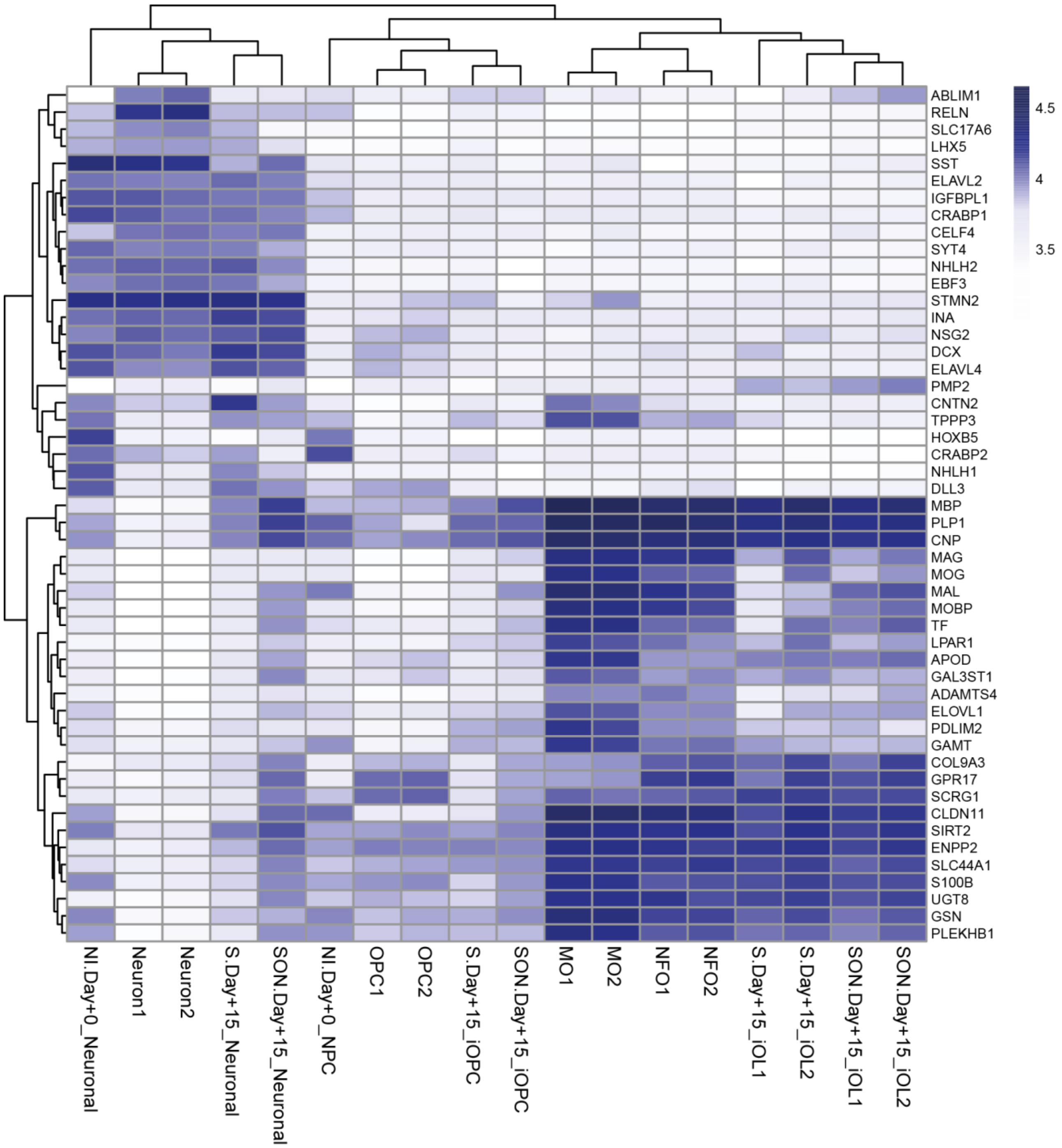

Figure S3. 50 most variable genes from co-clustering of iOPCs and iOLs with primary cells. Related to Figure 3.

Comparison of transcriptomes of hiPSC-derived cells with primary murine cells (Zhang et al., 2014). Expression heatmap of the 50 most variable genes in the analysis. Unsupervised hierarchical clustering based on Manhattan distances was performed on samples and genes and used for sorting of the heatmap. Abbreviations for primary reference samples: MO, myelinating oligodendrocytes; NFO, newly formed oligodendrocytes; OPC, oligodendrocyte precursor cells.

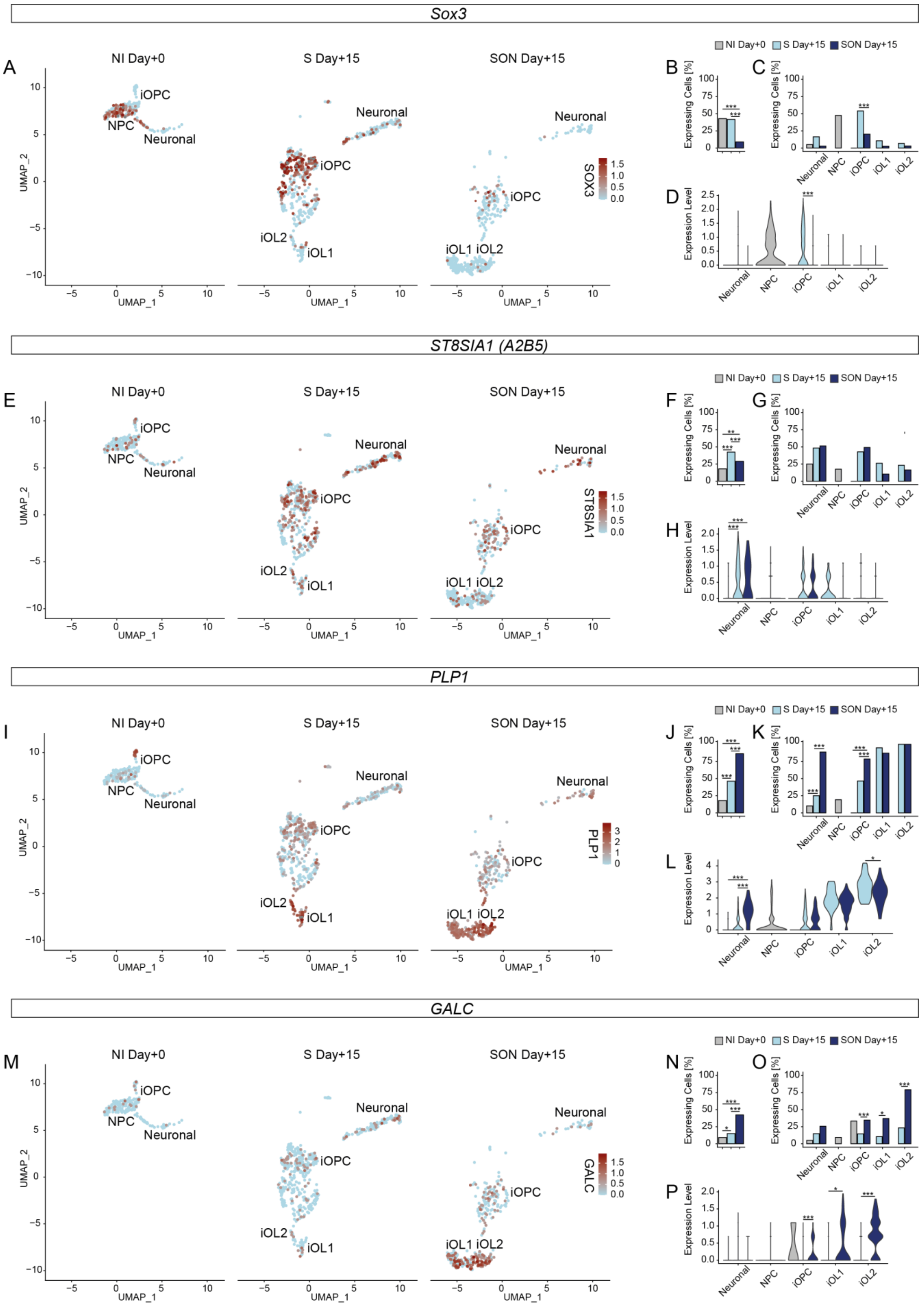
Sox3
NI Day+0
S Day+15
SON Day+15
NI Day+0 S Day+15 SON Day+15
A
B
C
D
iOPC
NPC
Neuronal
iOL1
iOL2
UMAP_1
UMAP_2
SOX3
Expressing Cells [%]
Expression Level
ST8SIA1 (A2B5)
E
F
G
H
ST8SIA1
PLP1
I
J
K
L
GALC
M
N
O
P

Figure S4. Illustration of extended marker gene expression from scRNAseq. Related to Figure 4.
(**A, E, I, M**) UMAP representation of selected marker genes (**A**) *SOX3*, (**E**) *ST8SIA1* (also known as A2B5), (**I**) *PLP1*, (**M**) *GALC* from scRNAseq of baseline sample after neural induction (NI Day+0, n = 217 cells, one independent experiment) and samples after 15 days of directed differentiation by SOX10 (S Day+15, n = 577 cells, one independent experiment) and SOX10-OLIG2-NKX6.2 (SON Day+15, n = 400 cells, one independent experiment) with annotated cell clusters of neural precursor cells (NPC), neuronal cells, induced oligodendrocyte precursor cells (iOPCs), induced oligodendrocyte cluster 1 (iOL1) and induced oligodendrocyte cluster 2 (iOL2). Expression value per cell plotted according to the colour intensity of the respective scale bar as indicated.
(**B, F, J, N**) Bar plots illustrate the abundance of expressing cells for each sample. * (p-value < 0.05), ** (p-value < 0.01), *** (p-value < 0.001) based on Fisher's exact tests (details provided in Table S5).
(**C, G, K, O**) Bar plots illustrate the abundance of expressing cells for each cluster split by sample. * (p-value < 0.05), ** (p-value < 0.01), *** (p-value < 0.001) based on Fisher's exact tests (details provided in Table S5).
(**D, H, L, P**) Violin plots of the normalized expression per sample within the respective cell type cluster. * (p-value < 0.05), ** (p-value < 0.01), *** (p-value < 0.001) based on Wilcoxon signed-rank test (details provided in Table S7).

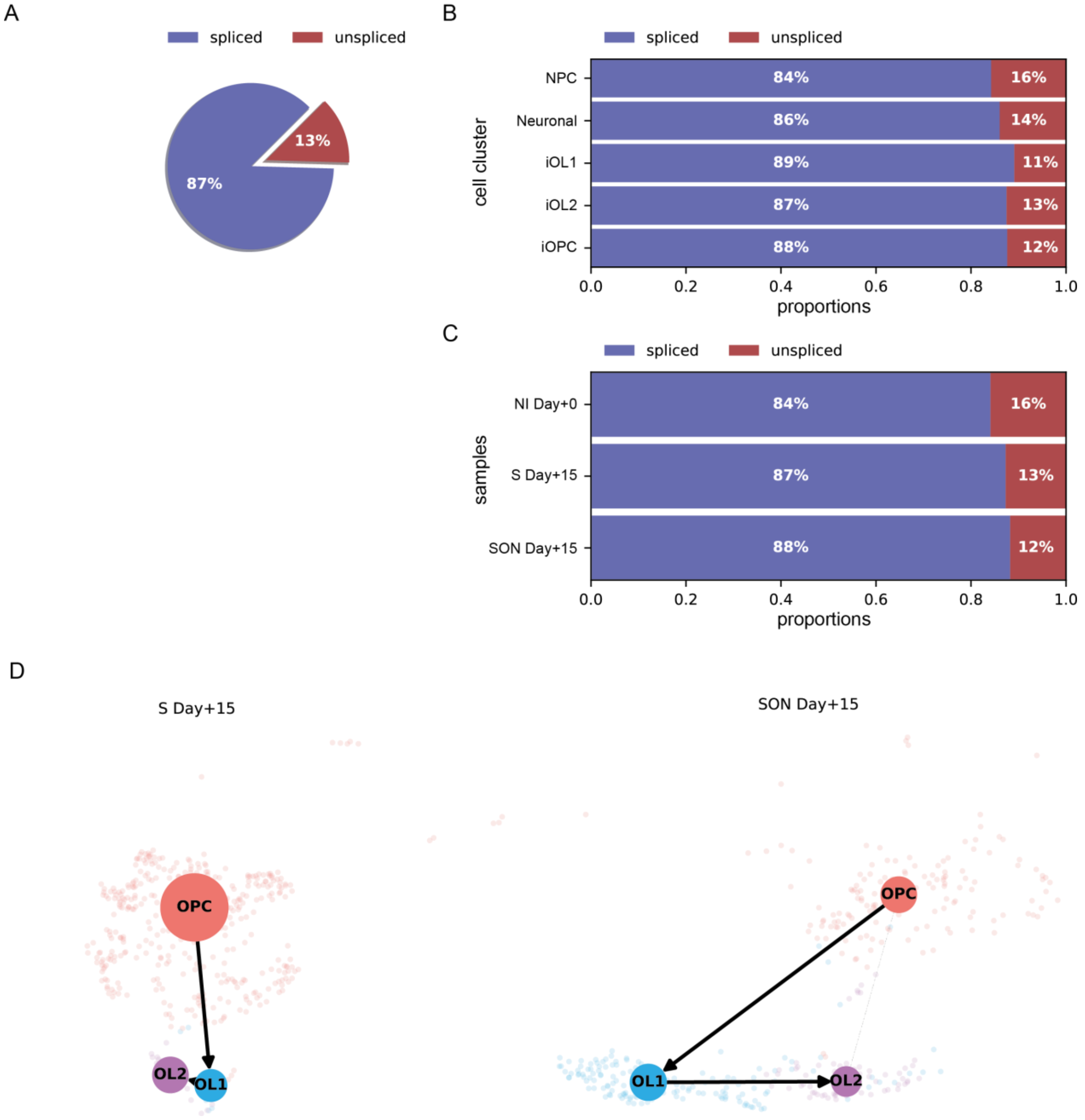

Figure S5. Ratio of unspliced/spliced mRNA variants and sample-independent main differentiation trajectory analysis. Related to Figure 5.

(A) Overall ratio of spliced vs. unspliced mRNA

(B) Cluster-dependent ratio of spliced vs. unspliced mRNA

(C) Sample-dependent ratio of spliced vs. unspliced mRNA

(D) Sample independent main differentiation trajectory analysis using Partition-based graph abstraction (PAGA) to illustrate cluster-to-cluster transitions within the S- and SON-promoted oligodendroglial differentiation.

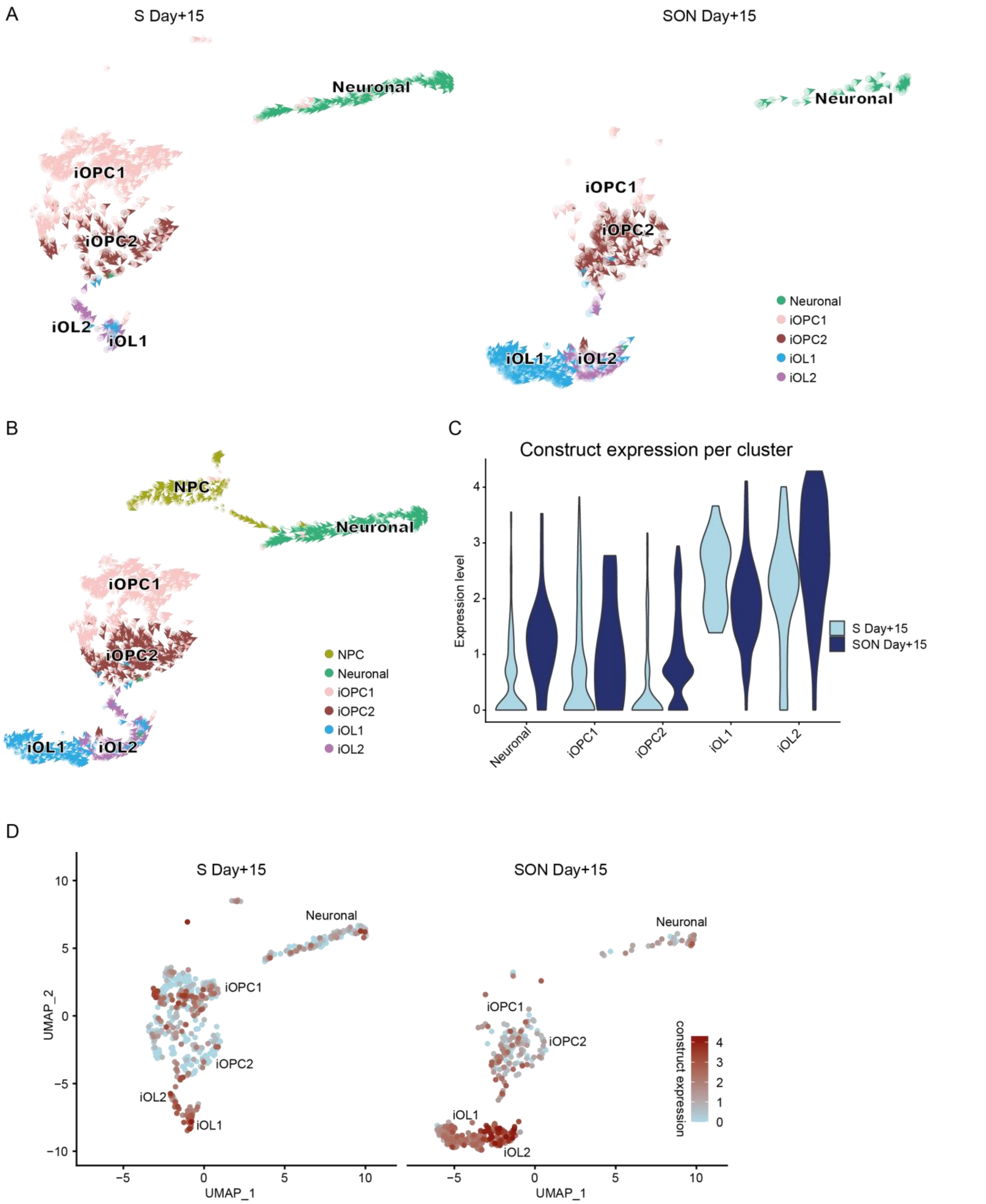

Figure S6. Differences of RNAvelocity between iOPC1 and iOPC2 are not based on different expression of directing TFs. Related to Figure 6.

(A) RNA velocity vectors projected on the single-cell UMAP-based dimension plot, indicating direction and speed of individual cells, including iOPC1 and iOPC2, in transcriptional space.

(B) Pooled Illustration of scVelocity of NI-, S- and SON-derived samples (NI Day+0, S Day+15, SON Day +15) including iOPC1 and iOPC2.

(C) Violin plots of normalized construct expression in Neuronal, iOPC1, iOPC2, iOL1 and iOL2 clusters split by S- and SON-sample (S Day+15, SON Day +15).

(D) Illustration of lentiviral construct expression from scRNAseq with UMAP dimension plot for S Day+15 and SON Day+15 with the normalized expression level indicated by colour. Related to Figure S6C.

Table S1. hiPSC lines in this study.

hiPSC cell line	ID LMU	Donor age	Donor sex	Passage
hiPSC donor 0	PSYLMUi001-A	27	male	P17
hiPSC donor 1	PSYLMUi002-A	24	male	P18
hiPSC donor 2	PSYLMUi003-A	19	male	P17
hiPSC donor 3	PSYLMUi004-A	31	male	P18
hiPSC donor 4	PSYLMUi006-A	52	male	P21
hiPSC donor 5	PSYLMUi023-A	47	male	P19
hiPSC donor 6	PSYLMUi027-A	53	male	P15

Table S2. Media, media supplements for cell cultivation and coating.

Item	Supplier	Cat.No.
iPS-Brew	Miltenyi Biotec	130-104-368
mTeSR1	Stem Cell	85850
ProFreeze CDM	Lonza	BEBP12-769E
DMEM/F-12 with GlutaMAX™ supplement	ThermoFisherScientific	31331028
N-2 Supplement (100x)	ThermoFisherScientific	17502048
B-27® Supplement (50X), without vitamin A	ThermoFisherScientific	12587010
Non-Essential Amino Acids Solution (NEAA)	ThermoFisherScientific	11140035
ß-Mercaptoethanol	ThermoFisherScientific	21985023
Insulin solution human	Sigma	I9278-5ML
SB431542	StemCell	72232
LDN193189 (HCl) - superstock	StemCell	72147
Retinoic acid (RA)	Sigma	R2625-50MG
SAG	Millipore	566660
PDGFaa	PeproTech	100-13A
IGF1	PeproTech	100-11
HGF	PeproTech	100-39
NT3	Peprotech	AF-450-03
Biotin	Sigma	B4639-100MG
dbcAMP	Sigma	D0627-250MG
T3	Sigma	T6397-100MG
Doxycycline	Clontech	NC0424034
Puromycin	ThermoFisherScientific	A1113803
ROCK-Inhibitor (Y-27632)	Selleckchem	S 1049
RevitaCell	ThermoFisherScientific	A2644501
Vitronectin	ThermoFisherScientific	A14700
Matrigel	BD Bioscience	354277
Poly-L-ornithine solution	Sigma	P4957
Laminin (mouse)	Sigma	T6397-100MG
EDTA	ThermoFisherScientific	15575-020
Accutase	Sigma	A6964
Anti-O4 microbeads	Miltenyi Biotec	130-096-670

Table S3. List of primary antibodies for immunostainings. Related to Figure 1, 2, 6, S1, S2.

Antigen	Dilution	Supplier	Cat.No.	Serotype	Host
O4	1:200	R&D Systems	MAB1326	IgM, monoclonal	mouse
MBP	1:50	Millipore	AB9348	IgG, polyclonal	chicken
MBP	1:200	Abcam	ab40390	IgG, polyclonal	rat

Pax 6	1:500	Millipore	AB2237	IgG, polyclonal	rabbit
Olig2 (H-10)	1:100	Santa Cruz	sc-515947	IgG, monoclonal	mouse
Flag-M2	1:100	Sigma	3165	IgG, monoclonal	mouse

Table S4: Abundance of cell clusters, gene marker expressing cells per samples and cluster. Provided separately as Excel file. Related to Figure 3, 4 and S4. Provided separately as Excel file.

Table S5: Statistical analysis of cell clusters abundance per sample, abundance of gene expressing cells by sample and abundance of gene expressing cells per cluster split by sample. Related to Figure 3, 4 and S4. Provided separately as Excel file.

Table S6: Average expression of cell clusters. Related to Figure 3, 4 and S4. Provided separately as Excel file.

Table S7: Statistical analysis of normalized gene expression by cell cluster and by cell cluster split by sample. Related to Figure 3, 4 and S4. Provided separately as Excel file.

Table S8: Differential gene expression between iOPC1 and iOPC2. Related to Figure 6. Provided separately as Excel file.

Table S9: Hypergeometric gene ontology term enrichment analysis of iOPC1 and iOPC2. Related to Figure 6. Provided separately as Excel file.

Supplemental References

Zhang, Y., Chen, K., Sloan, S.A., Bennett, M.L., Scholze, A.R., O'Keeffe, S., Phatnani, H.P., Guarnieri, P., Caneda, C., Ruderisch, N., et al. (2014). An RNA-sequencing transcriptome and splicing database of glia, neurons, and vascular cells of the cerebral cortex. J. Neurosci. Off. J. Soc. Neurosci. *34*, 11929–11947.
