# Supplementary figures and images for: Expression of Lineage Transcription Factors Identifies Differences in Transition States of Induced Human Oligodendrocyte Differentiation
Source: Cells. 2022 Jan 11;11(2):241. doi: 10.3390/cells11020241 (PMC8773672; doi:10.3390/cells11020241)

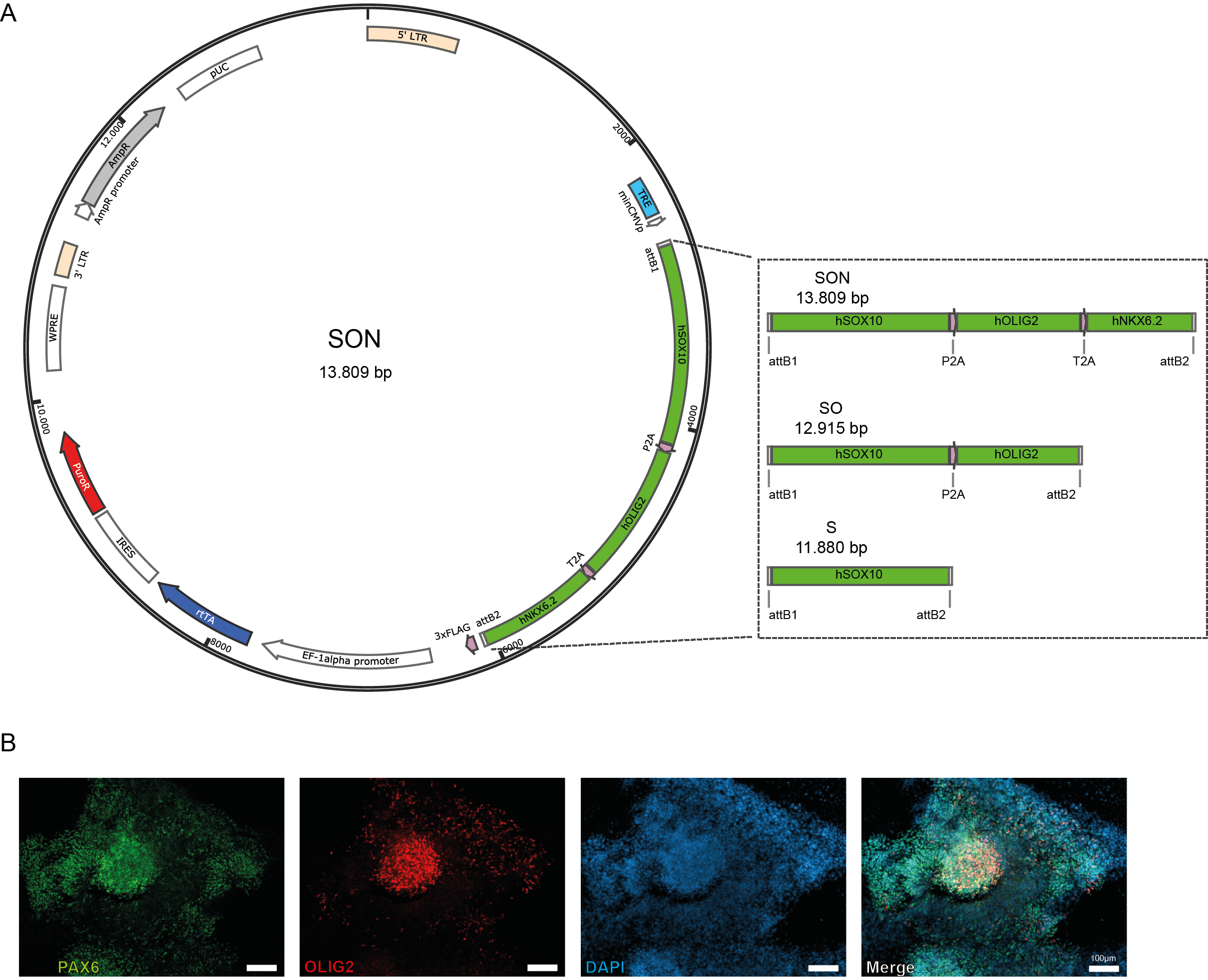

Supplement: Supplementary file 1 [file cells-11-00241-s001.zip › cells-1542523-supplementary/Figure S1.tif]

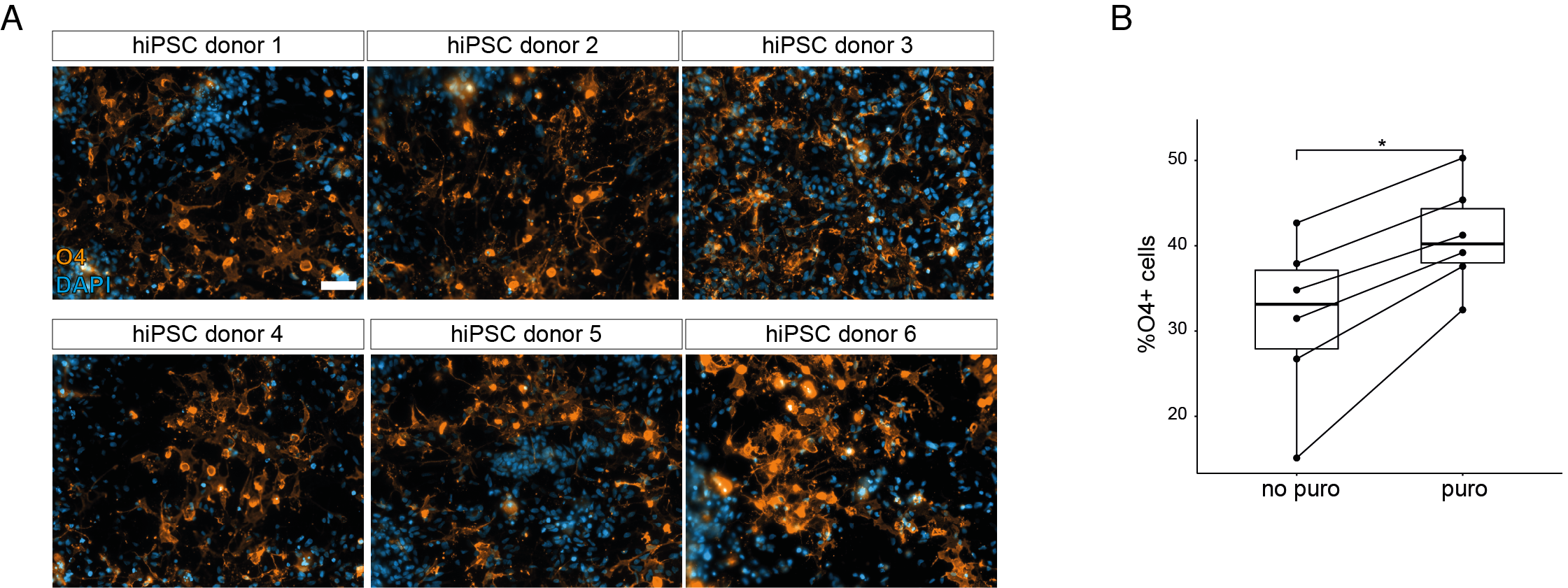

Supplement: Supplementary file 1 [file cells-11-00241-s001.zip › cells-1542523-supplementary/Figure S2.tif]

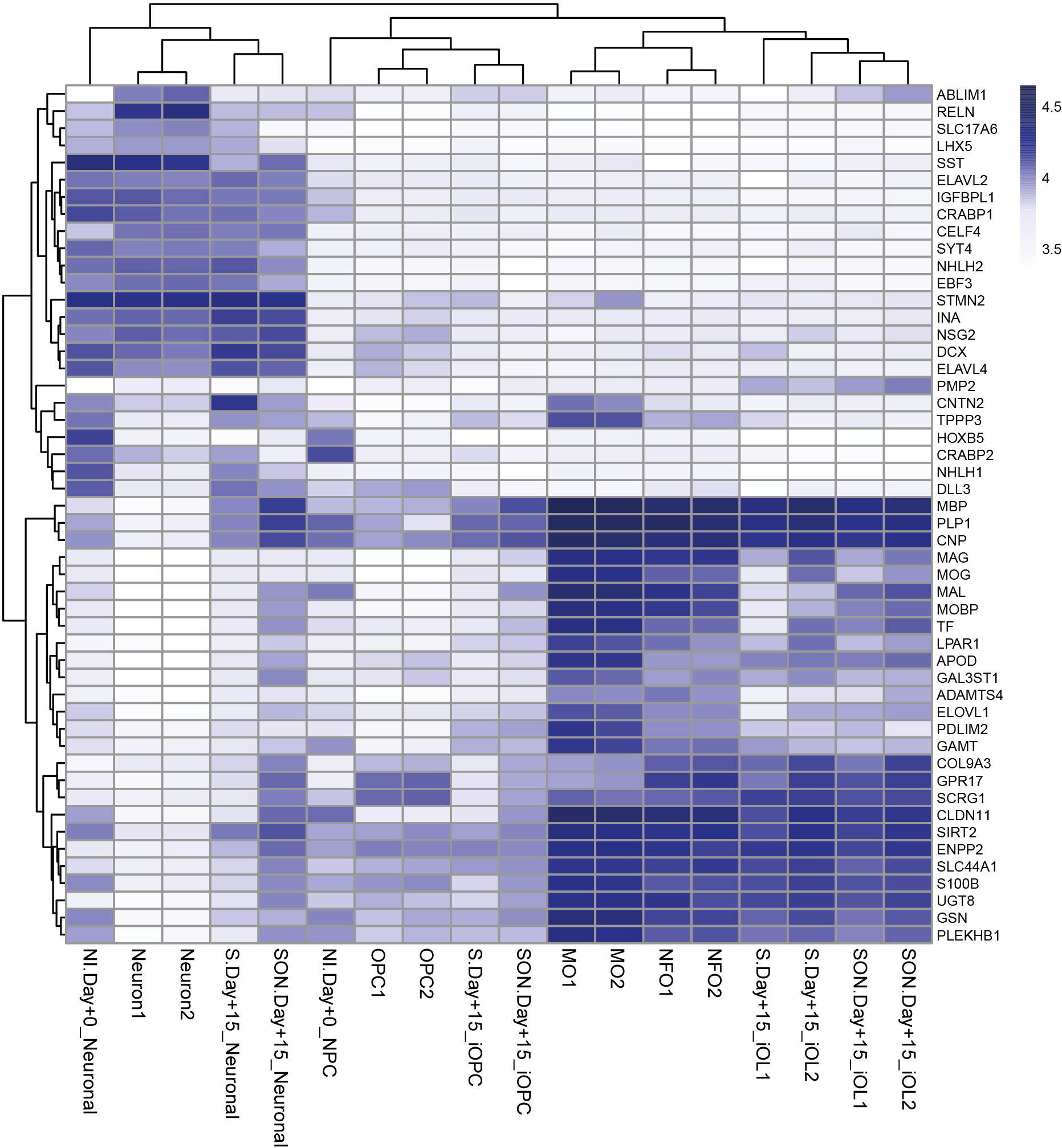

Supplement: Supplementary file 1 [file cells-11-00241-s001.zip › cells-1542523-supplementary/Figure S3.tif]

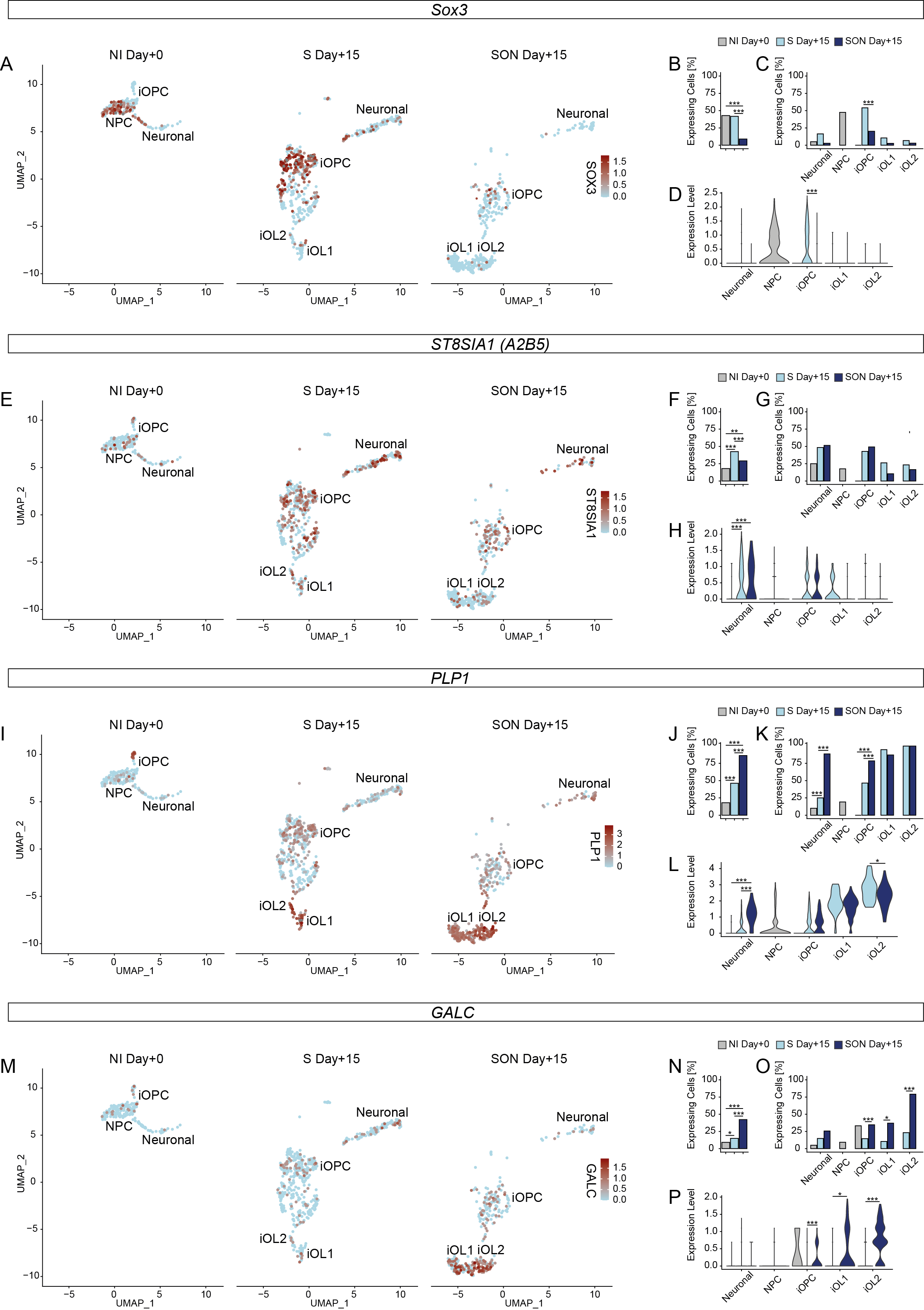

Supplement: Supplementary file 1 [file cells-11-00241-s001.zip › cells-1542523-supplementary/Figure S4.tif]

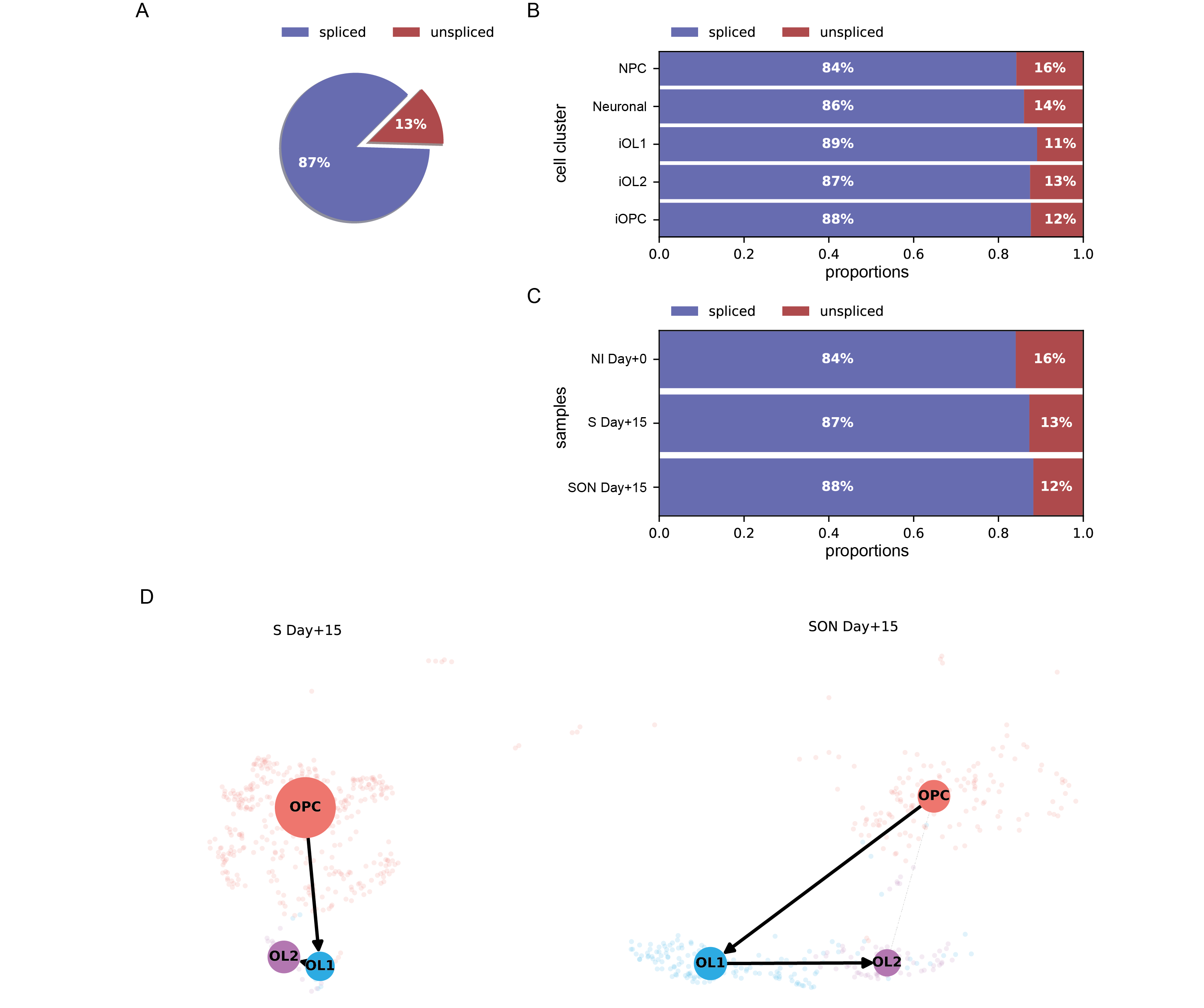

Supplement: Supplementary file 1 [file cells-11-00241-s001.zip › cells-1542523-supplementary/Figure S5.tif]

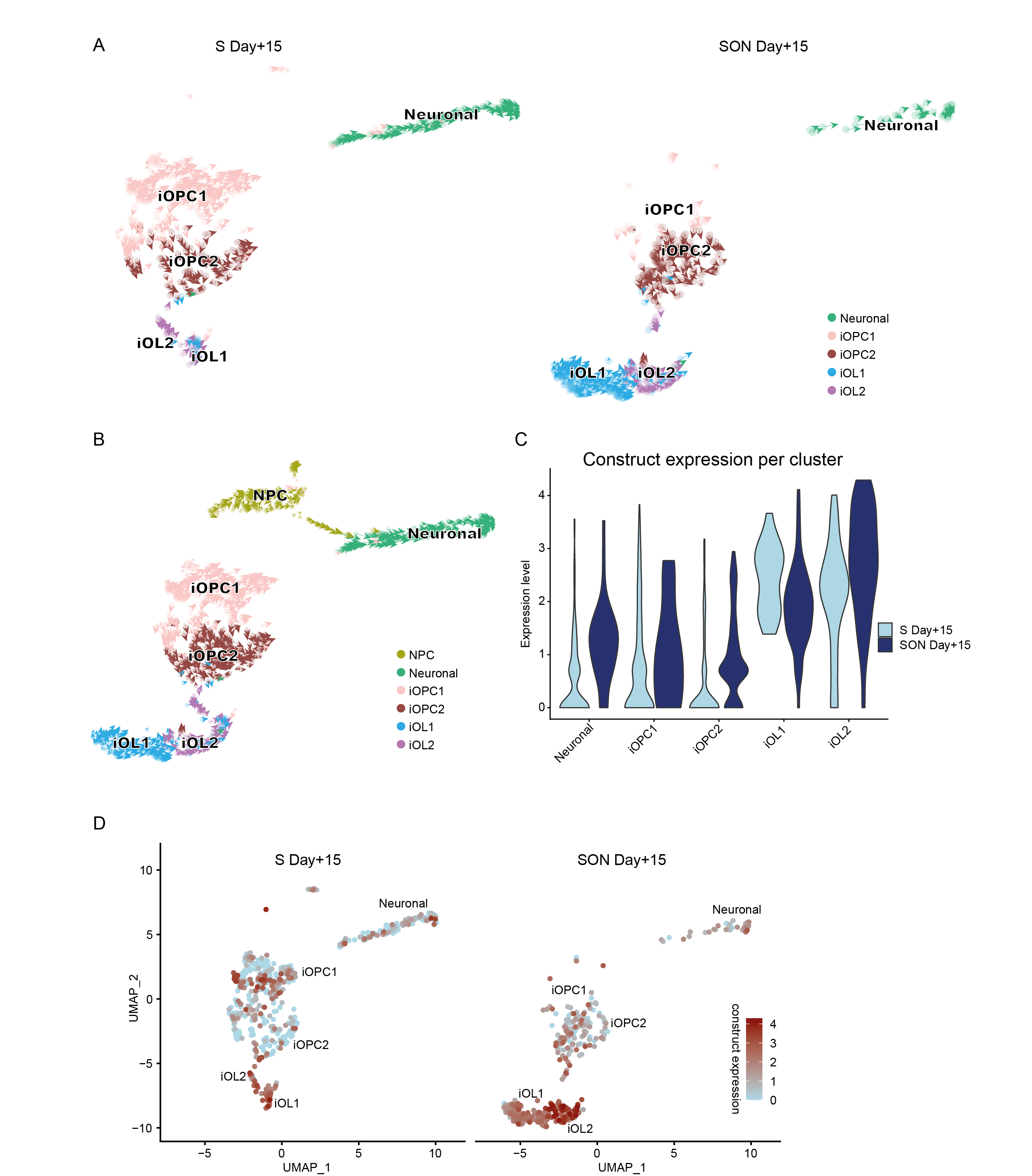

Supplement: Supplementary file 1 [file cells-11-00241-s001.zip › cells-1542523-supplementary/Figure S6.tif]
